# Predictors of Patients’ Intention to Interact With Doctors in Web-Based Health Communities in China: Cross-Sectional Study

**DOI:** 10.2196/13693

**Published:** 2019-06-07

**Authors:** Tailai Wu, Zhaohua Deng, Zhuo Chen, Donglan Zhang, Ruoxi Wang, Xiang Wu

**Affiliations:** 1 School of Medicine and Health Management Tongji Medical College Huazhong University of Science and Technology Wuhan China; 2 Department of Health Policy and Management College of Public Health University of Georgia Athens, GA United States; 3 School of Economics, Faculty of Humanities and Social Sciences University of Nottingham Ningbo China Ningbo China

**Keywords:** medical informatics, telemedicine, patients, physicians, community network, psychological theory, social theory

## Abstract

**Background:**

Web-based health communities provide opportunities for doctors and patients to interact with each other and change the traditional communication mode between doctors and patients. However, little is known about the predictors of patients’ intention to interact with doctors in Web-based health communities in China.

**Objective:**

The purpose of this study was to investigate what are the predictors of patients’ intention to interact with doctors in Web-based health communities in China.

**Methods:**

On the basis of two-factor theory and service convenience theory, we propose that the attributes of Web-based health communities including ease of use and perceived synchronicity influence patients’ intention to interact through convenience of Web-based health communities, whereas the attributes of physical health facilities such as inaccessibility and discontinuity affect patients’ intention to interact through inconvenience of physical health facilities. We employed the survey method to validate our hypothesized relationships. Through developing the measurement instruments, we collected 334 valid answers from Web health community users and utilized partial least square to analyze the data.

**Results:**

Ease of use (*t*_311_=2.924, *P*=.004) and perceived synchronicity (*t*_311_=2.353, *P*=.019) were found to influence convenience of Web-based health communities significantly, whereas inaccessibility (*t*_311_=3.189, *P*=.002) and discontinuity (*t*_311_=3.149, *P*=.002) were found to impact inconvenience of physical health facilities significantly. Meanwhile, both convenience of Web-based health communities (*t*_311_=2.353, *P*=.019) and inconvenience of physical health facilities (*t*_311_=2.787, *P*=.006) were found to affect patients’ intention to interact with doctors in Web-based health communities significantly. Therefore, all the proposed hypotheses were supported.

**Conclusions:**

Through including factors from both Web-based health communities and physical health facilities, we can understand patients’ intention to interact comprehensively. This study not only contributes to literature of doctor-patient interaction and Web-based health platforms but also provides implications to promote doctor-patient interaction online and offline.

## Introduction

### Background

Patients in China are increasingly using internet to understand and treat their health issues. Almost 32.7% Chinese internet users have sought support online for their health issues, and the size of Chinese internet health care market is expected to reach 90 billion RMB [1]. Meanwhile, Chinese doctors spent more than 50% of their online time to do health-related activities [2]. Among all the internet applications, Web-based health communities provide the opportunities for interaction between patients and doctors in China [3].

Web-based health communities allow patients to establish discussion groups with doctors and other patients who have common interest in certain health problems and provide online tools for patients to contact doctors directly [4]. Patients can interact with any doctors they prefer in Web-based health communities. Therefore, communication mode between patients and doctors have been changed from single direction communication to multiple direction communication [5]. In the meantime, interactions between doctors and patients have been shown to promote patients’ healthy behaviors and health information seeking, which could enhance their health status and alleviate the severity of many diseases including diabetes, hypertension, stroke, and Alzheimer disease [6,7]. Therefore, promoting interaction between patients and doctors in Web-based health communities is important for patients’ health. Previous literature has studied the factors that influence interaction between patients and doctors. For example, Schillinger et al [8] found functional health literacy had positive association with quality of physician–patient communication among diabetes patients. Hsu et al [9] showed doctors’ use of computers associated with patients’ general satisfaction, communication about medical issues, and comprehension of medical decisions made. Wald et al [10] revealed that the use of internet could lead to more informed patients and in turn better health care choices. Roter et al [11] investigated the role of gender in physician-patient interaction by using meta-analysis method and found that female physicians engaged in communication with patients longer than male physicians. Schouten and Meeuwesen [12] showed doctors might behave differently toward different ethnic patients in doctor-patient communication. Although previous literature has studied some predictors of interaction between doctors and patients, few of them considers the context of Web-based health communities and related factors.

Meanwhile, considering the large population and unique health care system in China, the role of Web-based health communities in China may be different [13]. In addition, special cultural, social, and institutional forces in China may make patients’ interaction with doctors in Web-based health communities distinct [14]. Thus, it is necessary to study patients’ interaction with doctors in Web-based health communities in China context. Therefore, we propose our research questions as follows: *What are the predictors of patients’ intention to interact with doctors in Web-based health communities in China?*

To address the research questions, we arrange the following sections as follows: we first apply the two-factor theory and service convenience theory to explore the predictors of patients’ intention to interact with doctors and their underlying mechanisms. Afterwards, we establish our research model by developing corresponding hypotheses. Methods and Results follow. Discussion, implications, and future directions are in the last section.

### Theoretical Foundation

Service convenience theory provides us the main theoretical perspective to understand patients’ interaction and the basis to construct the working mechanisms of patients’ interaction intention’s predictors. Meanwhile, based on two-factor theory, we can decide the predictors of patients’ intention to interact with doctors in Web-based health communities.

Service convenience theory assumes that consumers’ perceived convenience is influenced by different characteristics of service, firm-related factors, and individual consumer differences, and it also determines their evaluation of services [15]. Characteristics of service are the main determinants of perceived convenience. Given both Web-based health communities and physical health facilities provide the place for doctors to provide services to patients, they may affect the quality of doctors’ service. Therefore, it is possible to use service convenience theory to formulate the underlying mechanisms of predictors. To be specific, we propose convenience of Web-based health communities, and inconvenience of physical health facilities serve as the mechanisms for predictors of patients’ interaction.

Two-factor theory is originally used to understand satisfaction in organization context [16]. This theory suggests that there are 2 categories of factors that influence satisfaction: hygiene and motivation factors. Hygiene factors may lead to dissatisfaction when not present, whereas motivation factors can lead to satisfaction when present. Therefore, convenience of Web-based health communities and inconvenience of physical health facilities could be understood by two-factor theory as convenience and inconvenience could correspond to hygiene and motivation factors. Toward motivation factors, the attributes of Web-based health communities could be the sources, whereas attributes of physical health facilities could link to the hygiene factors.

### Research Model and Hypotheses Development

**Figure 1:**
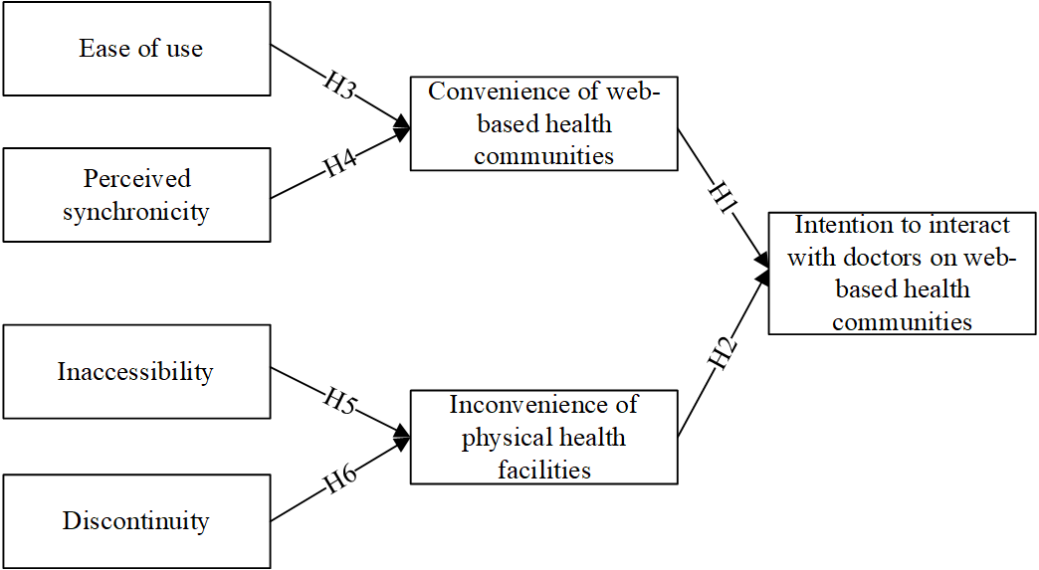
Research model and hypothesized relationships.

On the basis of service convenience theory, we propose that convenience of Web-based health communities and inconvenience of physical health facilities influence patients’ intention to interact with doctors in Web-based health communities. Meanwhile, according to two-factor theory, we hypothesize that ease of use and perceived synchronicity affect convenience of Web-based health communities, whereas inaccessibility and discontinuity impact inconvenience of physical health facilities. The assumed relationships are depicted in Figure 1.

### Convenience, Inconvenience, and Intention to Interact

Convenience can be defined as people’s perception of time and effort involved in performing some tasks [15]. Therefore, patients feel convenient when they use less time and effort in using Web-based health communities, whereas patient perceive inconvenience when physical health facilities cost them too much time and effort. Convenience have been shown to facilitate the adoption and use of different information technologies [17,18]. As convenience of Web-based health communities can give value to patients through offering medical resources directly to patients and responding patients’ health demands quickly [19], the value from Web-based health communities make patients adopt and use Web-based health communities. Meanwhile, inconvenience of physical health facilities may bring barriers or even threats to patients’ health problems solving. Thus, the barriers or threats from inconvenience of physical health facilities may make patients avoid using physical health facilities and switch to Web-based health communities. On the basis of the above reasoning and explanations, we hypothesize that:

- H1: Convenience of Web-based health communities influences patients’ intention to interact with doctors in Web-based health communities positively.
- H2: Inconvenience of physical health facilities influences patients’ intention to interact with doctors in Web-based health communities positively.

### Ease of Use, Perceived Synchronicity, and Convenience of Online Health Communities

Ease of use is the degree to which using Web-based health communities is free of effort [20]. Thus, ease of use of Web-based health communities may make patients cost little effort in using Web-based health communities and lead to patient feel using Web-based health communities be convenient. Previous literature has also shown the relationship between ease of use and convenience. For example, Yoon and Kim [18] showed that perceived ease of use positively influenced convenience of wireless LAN. Lai and Chang [21] found that perceived ease of use affected convenience of electronic book readers positively. Therefore, we can hypothesize that:

- H3: Ease of use influences convenience of Web-based health communities positively.

Perceived synchronicity is identified as 1 component of interactivity and defined as the degree to which the reception of messages is simultaneous to their sending in communication [22]. When patients can receive the feedback from doctors quickly in Web-based health communities no matter how far away they are, they will save their time and effort. Thus, patients may perceive convenience of using Web-based health communities. As such, we hypothesize that:

- H4: Perceived synchronicity influences convenience of Web-based health communities positively.

### Inaccessibility, Discontinuity, and Inconvenience of Physical Health Facilities

Inaccessibility is the opposite of accessibility and refers to the degree to which patients cannot use health care service to achieve the best health outcomes [23]. Inaccessibility contains several dimensions including unaffordability, physical inaccessibility, unacceptability of services, and inadequacy of service supply [24]. Inaccessibility of health care services in physical health facilities becomes the barriers for patients to access the health care services they need and cost patients much time and effort in physical health facilities. Therefore, inaccessibility of health care services in physical health facilities may cause inconvenience for patients. Afterwards, we can hypothesize that:

- H5: Inaccessibility influences inconvenience of physical health facilities positively.

Discontinuity is the degree to which the whole health care process for patients is not coherent and connected [25]. It also includes some aspects such as informational discontinuity, management discontinuity, and relational discontinuity. Discontinuity may go against building trust and bond between doctors and patients, and afterwards the compliance toward doctors’ medical treatment and advices [26]. Patients need to spend more time and effort to use discontinuous health care services. Thus, discontinuity of health care services in physical health facilities may also lead to inconvenience for patients. Afterwards, we can hypothesize that:

- H6: Discontinuity influences inconvenience of physical health facilities positively.

## Methods

### Measurement Instruments

To verify the proposed relationship in this study, survey method is employed in this study. Measurement instruments are developed based on previous literature. Items for intention to interact with doctors are adapted from Jang et al [27], items for convenience of Web-based health communities and inconvenience of physical health facilities are adapted from Yoon and Kim [18], items for ease of use are from Venkatesh and Davis [28], items for perceived synchronicity are adapted from Liu [29], and items for inaccessibility and discontinuity are adapted from Wang and Haggerty [30]. Besides the constructs studied in our research model, we also consider several control variables, which are mentioned in previous literature including age, gender, education, intensity and length of using Web-based health communities, and perceived health [7]. Meanwhile, the experience of filling online questionnaires of respondents is included in the questionnaire to test the non-naïve effect [31]. All items were measured by 5-point Likert scales, which are anchored from “1=strongly disagree” to “5=strongly agree.”

Given our measurement instrument was adapted and developed from English literature, back translation method is used to translate it into Chinese. One of the bilingual authors first translated the English version of measurement instrument into Chinese version. Afterwards, another bilingual author back translated the Chinese version into English version. The 2 authors checked the consistency between the 2 English versions by comparing them. Inconsistency is resolved through discussion between the 2 authors. After the translation, we have checked the quality of measurement instrument preliminarily by conducting a pretest through interviewing 9 experts in the area of medical informatics, health management, and 16 users of Web-based health communities. Through the pretest, the experts and users provide comments and suggestions to improve the quality of our measurement instrument. On the basis of the comments and suggestions, we decide our measurement instrument and present it in Multimedia Appendix 1.

### Data Collection

Given China has the largest population of internet users, we collected data from China [32]. To access to users of Web-based health communities efficiently, we employed the paid survey service from a leading online market research company. The service of market research firms allows for the efficiently administering online surveys and recruiting voluntary, motivated, and willing research participants for different research purposes [33]. This company has a sampling base that contained 2.6 million members, and more than 1 million of them were the daily questionnaire respondents. Given the objective of this study is to investigate patients’ intention to interact with doctors in Web-based health communities, respondents with experience of interacting with doctors in Web-based health communities were randomly invited to fill out our questionnaires randomly from the sample base. The institutional review board of Tongji Medical College, Huazhong University of Science and Technology has approved our study procedures (No 2017S319). Through 3 weeks of deployment, we received a total of 427 responses.

To ensure the data quality and reduce social desirability bias, several actions are taken during the data collection. First, attention-trap and reverse-coded questions were used in the questionnaire to reduce single-method bias and check whether respondents were reading all questions fully and honestly. Second, several screening questions were set to check whether the respondents were patients who interacted with doctors in Web-based health communities such as whether the respondents had interacted with doctors in Web-based health communities, which Web-based health communities’ respondents used most, and whether respondents were the members of Web-based health communities. Finally, the cases with missing values or similar values for all questions were discarded. Almost 93 answers were excluded, which leaves 334 valid responses. Therefore, the percent of invalid responses is 27.8% (93/334), which is reasonable [34].

## Results

### Demographic Information

The demographic information of our sample is showed in Table 1.

**Table 1:** Demographic information of sample participants.

Characteristics		n (%)
**Age (years)**		
	<25	61 (18.3)
	25-30	122 (36.5)
	>30	151 (45.2)
**Gender**		
	Male	138 (41.3)
	Female	196 (58.7)
**Education**		
	High school	7 (2.1)
	College	294 (88)
	Master’s degree and above	33 (9.9)
**Intensity of using Web-based health communities (hour/day)**		
	<0.5	274 (82)
	0.5-1	54 (16.2)
	>1	6 (1.8)
**Length of using Web-based health communities (years)**		
	<1	126 (37.7)
	1-5	164 (49.1)
	>5	44 (13.2)

### Reliability and Validity

To examine the reliability and validity of our measurement instrument, we conduct the confirmatory factor analysis by using SmartPLS 2.0.3M, which is a software developed by SmartPLS GmbH and is used for structural equation modeling [35]. Table 2 presents the results on reliability. The values of Cronbach alpha and composite reliabilities are all above .7; thus, confirming good reliability for all constructs [36]. Next, the values of average variance extracted (AVE) of each construct are all above 0.5, and loadings for each items are also all above 0.7; thus, reflecting good convergent validity [33]. Finally, Table 3 presents the results on validity. The values of square roots of AVEs are all greater than the inter-construct correlations, thus showing good discriminant validity [37]. Therefore, reliability and validity of our measurement instrument are acceptable.

Besides the tests of validity and reliability, common method bias was also considered. First, we went through the values of correlation coefficients among contracts in Table 3 to check whether they are too high (*r*>.90) and found all the values were not beyond the threshold. Second, we conducted Harman single factor test by the principle component analysis. A total of 7 factors were extracted, and the first extracted factor in the unrotated solution accounted for 23.53%, which is less than 50% [38]. Finally, we used marker variable technique to test the bias. Perceived organizational support was chosen as the marker variable as it was not relevant to our study [39]. The analysis result showed the average value of correlation coefficients between perceived organization support and other variables was only 0.156. Therefore, common method bias was not present in our study.

**Table 2:** Construct reliability and convergent validity.

Construct		Factor loadings	Composite reliability	Average variance extracted	Cronbach alpha
**Intention to interact (ITI)**					
	ITI1	0.8363	0.836	0.6301	.7088
	ITI2	0.7369	—^a^	—	—
	ITI3	0.805	—	—	—
**Convenience of Web-based health communities (CE)**					
	CE1	0.8641	0.8501	0.6556	.7388
	CE2	0.7124	—	—	—
	CE3	0.8443	—	—	—
**Inconvenience of physical health facilities (IC)**					
	IC1	0.8189	0.8309	0.6211	.7013
	IC2	0.7576	—	—	—
	IC3	0.7866	—	—	—
**Ease of use (EOU)**					
	EOU1	0.8101	0.8503	0.6543	.7376
	EOU2	0.8021	—	—	—
	EOU3	0.8145	—	—	—
**Perceived synchronicity (PSY)**					
	PSY1	0.7957	0.8392	0.635	.7128
	PSY2	0.8038	—	—	—
	PSY3	0.791	—	—	—
**Inaccessibility (IAY)**					
	IAY1	0.7462	0.844	0.5758	.7547
	IAY2	0.7044	—	—	—
	IAY3	0.8352	—	—	—
	IAY4	0.7435	—	—	—
**Discontinuity (DC)**					
	DC1	0.7442	0.834	0.5569	.7368
	DC2	0.7848	—	—	—
	DC3	0.7321	—	—	—
	DC4	0.7225	—	—	—

^a^—Not applicable.

**Table 3:** Discriminant validity.

	ITI^a^	CE^b^	IC^c^	EOU^d^	PSY^e^	IAY^f^	DC^g^
ITI	0.7938^h^	—^i^	—	—	—	—	—
CE	0.3484	0.8097^h^	—	—	—	—	—
IC	0.3826	0.2979	0.7881^h^	—	—	—	—
EOU	0.3477	0.4166	0.2576	0.8089^h^	—	—	—
PSY	0.3192	0.3642	0.2057	0.3835	0.7969^h^	—	—
IAY	0.2415	0.289	0.4002	0.1852	0.0593	0.7588^h^	—
DC	0.1245	0.2369	0.4039	0.1788	0.0991	0.3697	0.7463^h^

^a^ITI: intention to interact.

^b^CE: convenience of Web-based health communities.

^c^IC: inconvenience of physical health facilitates.

^d^EOU: ease of use.

^e^PSY: perceived synchronicity.

^f^IAY: inaccessibility.

^g^DC: discontinuity.

^h^The square roots of average variances extracted.

^i^—: not applicable.

### Analysis Results of Structural Model

**Figure 2:**
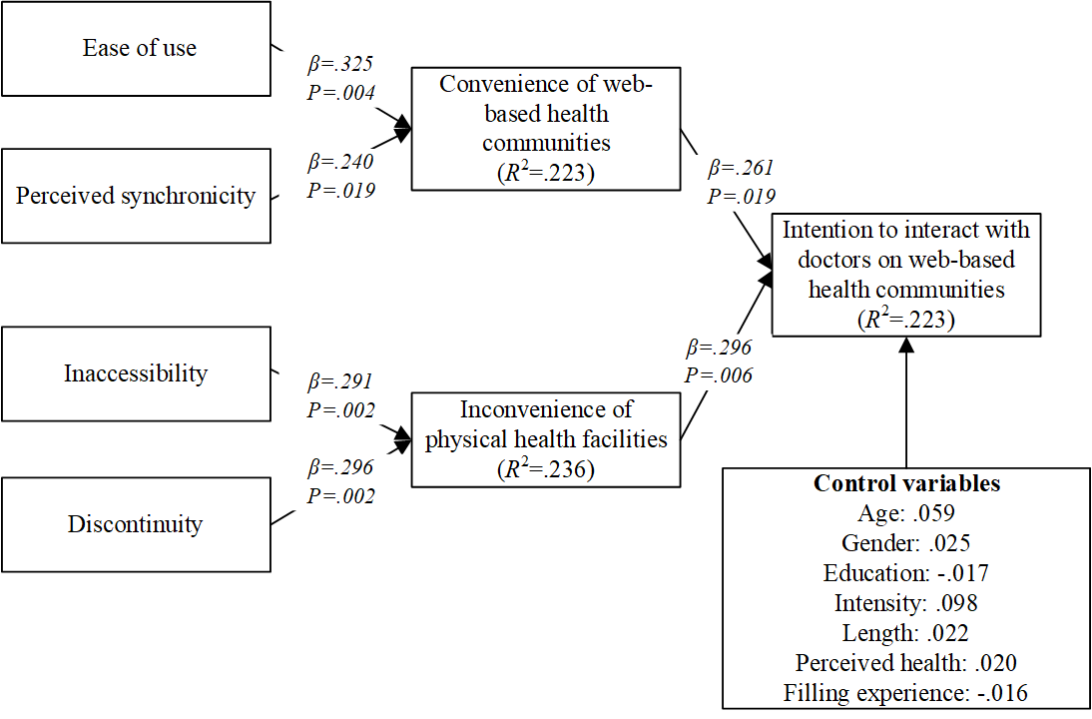
Analysis results of hypothesized model.

Through conducting the bootstrapping analysis in PLS, we test the hypothesized relationships in this study. The analysis results are shown in Figure 2. Both convenience of Web-based health communities and inconvenience of physical health facilities influence patients’ intention to interact with doctors in Web-based health communities. Therefore, H1 and H2 are supported. These results confirm the effectiveness of service convenience theory. Meanwhile, ease of use and perceived synchronicity are both shown to influence convenience of Web-based health communities. Therefore, H3 and H4 are supported. These results suggest the attributes of Web-based health communities do determine patients’ perception of Web-based health communities. Finally, inaccessibility and discontinuity are found to influence inconvenience of physical health facilities. Therefore, H5 and H6 are supported. There results reveal the attributes of physical health facilities also decide patients’ feeling of physical health facilities.

## Discussion

### Principal Findings

This study explores the predictors of patients’ intention to interact with doctors in Web-based health communities. On the basis of service convenience theory, we propose convenience of Web-based health communities and inconvenience of physical health facilities influence patients’ intention to interact with doctors in Web-based health communities directly. Meanwhile, based on two-factor theory, we propose that ease of use and perceived synchronicity influence convenience of Web-based health communities, whereas inaccessibility and discontinuity influence inconvenience of physical health facilities. All the proposed relationships are manifested by using the survey method. These results imply that our research model can help understand patients’ intention to interact with doctors in Web-based health communities adequately.

### Implications

This study provides both theoretical and practical implications. For the theoretical implications, we first integrate service convenience theory and two-factor theory to understand patients’ intention to interact with doctors in Web-based health communities. Through the integration, we can see patients’ intention to interact through a complement theoretical view. Second, we contribute to patient-doctor interaction literature by studying their predictors from patients’ view on Web health community context first. We consider the factors from Web-based health communities including ease of use and perceived synchronicity. Meanwhile, we also investigate the factors from physical health facilities such as inaccessibility and discontinuity. These factors answer our research questions directly. Finally, we explore the mechanisms of the predictors of patients’ intention to interact with doctors in Web-based health communities. The mechanisms help us improve our theoretical modeling of patients’ intention to interact.

Besides the theoretical implications, this study also provides its practical utility. First, this study confirms that Web-based health communities provide an important space for the interaction between doctors and patients. Second, there are implications for both health policy makers and Web health community managers. On the basis of factors from Web-based health communities, Web health community managers should provide enough tools and ensure the functionality of their communities for patients to communicate with doctors. Meanwhile, health policy makers should focus on the accessibility and continuity of physical health facilities, not just patients’ satisfaction. Third, the measure scales of convenience and inconvenience could be the direct indicators to reflect patients’ intentional or actual interaction with doctors in Web-based health communities. Finally, telehealth, which use the information communication technology to provide health care service distantly, have been developed in China for many years [40,41]. However, a recent study about telehealth in China has shown that the Chinese patients’ adoption of telehealth is quite low although they are aware of telehealth [42]. Therefore, to explore the drivers of telehealth usage is necessary. As Web-based health communities can take some form of telehealth, the factors explored in our study can be examined to promote the utilization of telehealth.

### Limitation and Future Direction

The limitations in this study can be the basis for future research directions. First, only 4 factors based on two-factor theory are included. On the basis of our current level of explained variance of convenience and inconvenience, more factors could be relevant. Second, although patients’ intention could link to their actual behavior, it is necessarily to study patients’ actual interaction with doctors in Web-based health communities as the actual behaviors can give more direct evidence of the effectiveness of our research model. Finally, we use cross-sectional data to study patients’ intention to interact and did not account the dynamic of variables in our model. A longitudinal study would be useful as a follow-up.

### Conclusions

This study examines the predictors of patients’ intention to interact with doctors in Web-based health communities based on service convenience theory and two-factor theory. Through including factors from both Web-based health communities and physical health facilities, we establish a theoretical model to understand patients’ intention to interact comprehensively. Through collecting data randomly from the sample pool of a research company, we validate the proposed research model. This study not only contributes to literatures of doctor-patient interaction and we health communities but also provides implications to promote doctor-patients interaction online and offline.

## Appendix

Multimedia Appendix 1 Measurement instrument.

## Abbreviations

AVE: average variance extracted
