# Supplementary material for: Predictors of Patients’ Intention to Interact With Doctors in Web-Based Health Communities in China: Cross-Sectional Study
Source: J Med Internet Res. 2019 Jun 7;21(6):e13693. doi: 10.2196/13693 (PMC6592481; doi:10.2196/13693)
Supplement: Multimedia Appendix 1 [file jmir_v21i6e13693_app1.pdf]

Appendix A. Measurement instrument

Table A. Measurement instrument	
Constructs	Items
Intention to interact with doctors	• I will exchange health information with doctors on online health platform<br>• I will response to doctors on online health platform quickly.<br>• I will interact with doctors on online health platform intensively.
Convenience of online health platforms	• It is convenience for me to interact with doctors by using this online health platform.<br>• Using online health platform gives me convenience in interacting with doctors.<br>• I find online health platform are convenient for interacting with doctors.
Inconvenience of physical health facilities	• It is not convenience for me to interact with doctors by using these online health platform.<br>• Physical health facilities do not give me convenience in interacting with doctors.<br>• I find physical health facilities are not convenient for interacting with doctors.
Ease of use	• My usage with the online health platform is clear and understandable.<br>• Using online health platform does not require a lot of my mental effort.<br>• I find the online health platform to be easy to use.
Perceived synchronicity	• Getting information from the online health platform is very fast.<br>• I was able to obtain the information I want without any delay through online health platform.

	• When I clicked on the links, I felt I was getting instantaneous information on the online health platform.
Inaccessibility	• It is not easy to get health advice from physical health care facilities.<br>• There is long distance to get to the physical health care facilities.<br>• It is long to wait for starting my consultation in physical health care facilities.<br>• I have times to find it difficult to get health care because I had to take time off work.
Discontinuity	• There is not a health professional knows me best at physical health facilities.<br>• In past 12 months, I was took care by different health person in physical health facilities.<br>• I have times that the person I was seeing did not have access to my recent tests or exam results in physical health facilities.<br>• I have times that the person I was consulting did not know my most recent medical history in physical health facilities.
